# Elevation Data Statistical Analysis and Maximum Likelihood Estimation-Based Vehicle Type Classification for 4D Millimeter-Wave Radar

**DOI:** 10.3390/s25092766

**Published:** 2025-04-27

**Authors:** Mengyuan Jing, Haiqing Liu, Fuyang Guo, Xiaolong Gong

**Affiliations:** School of Transportation and Logistic Engineering, Shandong Jiaotong University, Jinan 250357, China; mengyuanjing45@gmail.com (M.J.); fuyangguo4@gmail.com (F.G.); xiaolonggong17@gmail.com (X.G.)

**Keywords:** traffic monitoring, 4D millimeter-wave radar, elevation feature analysis, maximum likelihood estimation, vehicle classification

## Abstract

Traditional 3D radar can only detect the planar characteristic information of a target. Thus, it cannot describe its spatial geometric characteristics, which is critical for accurate vehicle classification. To overcome these limitations, this paper investigates elevation features using 4D millimeter-wave radar data and presents a maximum likelihood estimation (MLE)-based vehicle classification method. The elevation data collected by 4D radar from a real road scenario are applied for further analysis. By establishing radar coordinate systems and geodetic coordinate systems, the distribution feature of vehicles’ elevation is analyzed by spatial geometric transformation referring to the radar installation parameters, and a Gaussian-based probability distribution model is subsequently proposed. Further, the data-driven parameter optimization on likelihood probabilities of different vehicle samples is performed using a large-scale elevation dataset, and an MLE-based vehicle classification method is presented for identifying small and large vehicles. The experimental results show that there are significant differences in elevation distribution from two different vehicle types, where large vehicles exhibit a wider range of left-skewed distribution in different cross-sections, while small vehicles are more concentrated with a right-skewed distribution. The Gaussian-based MLE method achieves an accuracy of 92%, precision of 87% and recall of 98%, demonstrating excellent performance for traffic monitoring and related applications.

## 1. Introduction

The real-time collection of road traffic parameters and accurate evaluation of traffic congestion are essential for implementing effective strategies to alleviate congestion and enhance traffic flow [1,2,3]. Millimeter-wave radar is widely applied for detecting traffic flow in a downward-looking direction to obtain the vehicles’ trajectories, which can be used in target tracking, traffic parameter calculation, and security management [4]. The frequency-modulated continuous wave (FMCW) millimeter-wave radar has significant advantages, e.g., high measurement accuracy, for the low-speed targets, as well as multi-objective imaging quality with a high-resolution capacity, a wide detection range, and strong anti-interference capabilities [5]. With its anti-interference capability under different weather conditions such as rain and fog, the millimeter-wave radar has significant advantages in intelligent transportation systems compared with traditional traffic detectors like cameras, loop sensors, and geomagnetic detectors [6].

Traditional millimeter-wave radar can be classified as 3D radar that provides target information including the distance, target speed, and azimuth angle. The limitation is that it can only detect the planar characteristic information of the target on the road, while it cannot describe the spatial geometric characteristics of the target [7,8]. This feature does not affect the target tracking performance of the radar, but it leads to mediocre performance in target classification. Although many scholars have applied the radar cross-section (RCS) as the primary feature for target classification, the RCS value is generally fuzzy and dynamically changing in the complex road environment. The lack of features results in poor performance of traditional 3D radar in reliable and accurate vehicle classification, especially in a densely multi-target road scenario where the interference from adjacent vehicles is more severe [9,10].

Different from 3D radar, the 4D millimeter-wave radar can also detect the elevation of a target. This capability significantly improves spatial positioning accuracy, enhances dynamic target trajectory tracking, and provides new data support for the classification of available vehicle types. However, current research on the analysis and feature extraction of elevation data remains insufficient, and the elevation information contained in this kind of data has not been effectively utilized in the field of vehicle recognition and classification. Particularly, existing studies exhibit a critical gap in both generalized theoretical approaches and structured frameworks for processing, analyzing, and applying radar elevation data in a systematic manner. Consequently, the systematic statistical exploration of 4D radar elevation data for uncovering intrinsic distribution features of traffic targets in an actual road scenario is critical to formulating a theoretical framework for traffic detection applications.

This paper investigates elevation features using the data obtained by 4D millimeter-wave radar and presents a maximum likelihood estimation (MLE)-based vehicle classification method. The differences between small and large vehicles on statistical parameters, probability distributions, and skewness characteristics are analyzed and further used for type classification. This accomplishment can provide effective support for further intelligent transportation applications, such as fine-tuned traffic volume estimation and road capacity calculation.

## 2. Related Work

The comprehensiveness and precision of traffic detection are the prerequisites for modern transportation systems to function effectively. Currently, traffic parameters are primarily detected using inductive loops and geomagnetic devices [11,12], onboard floating devices [13], video image devices [14,15], and radar devices [16]. In particular, a radar detector identifies vehicle targets by the differences between the transmitting signal and the echo signal, and it calculates the vehicle speed based on the Doppler effect. The millimeter-wave radar generally works at a 30–300 GHz frequency band, and the wavelength is from 1 to 10 mm, which has significant advantages in high measurement accuracy for the low-speed targets, as well as multi-objective imaging quality with a high-resolution capacity, wide detection range and strong anti-interference capability [17]. Therefore, the millimeter-wave radar is widely applied in measuring traffic parameters, providing the needed traffic state estimation for congestion avoidance strategies to improve traffic efficiency in modern transportation systems.

Currently, the millimeter-wave radar is mainly used in target identification and trajectory tracking for traffic management and advanced driver-assistance vehicles. In [18], the authors present a calibration method for the motional frequency spread in wide-band FMCW automotive millimeter-wave radar. The proposed method improves radar measurement accuracy, making it more reliable for wide-range traffic monitoring in dynamic environments. In [19], the authors conduct an in-depth analysis of the perception accuracy of millimeter-wave radars in smart roads, verifying that these radars can accurately perceive traffic flow and vehicle speed, even in challenging environmental conditions, like adverse weather. In [20], an improved vehicle trajectory tracking model is proposed based on the Hungarian algorithm for millimeter-wave radar point cloud data at intersections. The model uses millimeter-wave radar data to accurately track vehicle movement. This enables the detection of vehicle speed and traffic flow in complex, congested intersections. In [21], the authors propose a multi-target measurement method for FMCW radar based on trapezoidal waveform modulation, incorporating TFBM and FGTC algorithms to optimize the ability to measure the range and velocity of multiple vehicles, which greatly enhances traffic flow monitoring in dense traffic areas. Referring to the aforementioned literature, the millimeter-wave radar is mainly applied in vehicle detection and trajectory tracking, providing more comprehensive and reliable data support for traffic management and control.

The echo signal from millimeter-wave radar also contains information about the shape of the target, which can be used to classify the vehicle. In [22], the histogram of the near-field RCS is calculated from the samples, and further, a nearest neighbor rule to classify conducting plates with different shapes based on their RCS histogram is proposed. Based on the achievements, the authors use several supervised machine learning and classification methods for further research where the deep learning network classifier performs better in accuracy than the traditional K-nearest neighbor method [23]. In [24], through extensive data analysis, it is found that the RCS distribution of the vehicles is between 75 dB and 100 dB. The RCS values of the small vehicle are concentrated with an average of 85.6 dB, while the large vehicle is concentrated with an average of 93.7 dB. The probability distributions for the two types of vehicles show certain distinctive characteristics that can be used for target classification. In [25], the target information is accumulated based on the changes in posture angles to generate RCS sequences, and statistical features are extracted. Further feature extraction is carried out through Melin transformation, and the results show that the average RCS values of the three categories of targets (buses, cars and pedestrians) show certain differences. The existing vehicle classification methods primarily achieve their purpose through the analysis of RCS data. Since the RCS describes the target’s reflection intensity characteristics and does not accurately capture the vehicle’s geometric shape, therefore, it makes it difficult to achieve accurate target classification based on RCS alone.

Different from the traditional millimeter-wave radar, the 4D radar not only provides information such as the target’s position, velocity and RCS but also provides elevation of a vehicle. Radar operates by periodically modulating the frequency of its transmitted signal, which varies linearly within a specific range. The radar emits this modulated signal and awaits its reflection from target objects. Upon receiving the reflected signal, the radar mixes it with the locally transmitted signal to generate an intermediate frequency signal. Through signal processing techniques such as Fast Fourier Transform (FFT), the system analyzes the frequency components to extract the target range and velocity information. Currently, the 4D radar is widely applied in real-time traffic monitoring and advanced driver-assistance systems (ADASs) for autonomous driving. In [26], the paper proposes a framework for testing radar-camera baseline fusion algorithms in a motorway roadside scenario using SUMO and CARLA simulators. In the framework, a roadside multi-sensor perception dataset is generated through co-simulation for deep-learning object detection under different weather and lighting conditions. In [27], the authors introduce a method for robust vehicle pose estimation by fusing 4D radar and camera data. An extended Kalman filter (EKF) is used to fuse heading angle and forward velocity by high-resolution 4D radar and the yaw rate obtained by a camera. The method exhibits good performance in foggy environments. In [28], a deep-learning-based 4D radar odometry method is proposed which uses coarse-to-fine optimization and a sliding window iteration to estimate vehicle pose. A feature extraction network handles sparse 4D radar data, while pose estimation is refined with motion information and regression. In [29], using an uncalibrated 4D millimeter-wave radar and a traffic monocular camera, the authors present an automatic coarse-to-fine calibration method based on double rotations of the position vectors for robust vehicle target detection in roadside installation scenario.

Since the elevation can be acquired by a 4D radar model, numerous scholars have conducted an analysis and exploration of using radar elevation data, achieving certain incremental research outcomes. In [30], the paper focuses on the application of elevation information in 4D radar datasets for autonomous driving. By integrating Doppler, range, azimuth and elevation data, the paper proposes a method that integrates elevation information to improve the accuracy of 3D object detection. Experimental results show that the 4D radar effectively improves the accuracy and robustness of object recognition. In [31], the elevation information together with Doppler, range and azimuth from 4D radar tensor (4DRT) data containing 35,000 frames is used for vehicle target detection under different road structures. The data of a 4D radar is combined with high-resolution LiDAR, stereo cameras and RTK-GPS, and a neural network is applied for the research. By incorporating elevation information, the 4D radar model significantly improves object detection accuracy and robustness, especially under complex weather conditions. These studies highlight the significant application of radar elevation data in traffic monitoring, particularly in vehicle detection under complex road environments. Hence, 4D radar elevation data provide additional effective information support and new application opportunities for Intelligent Transportation Systems (ITSs).

Meanwhile, the RCS is widely used in vehicle detection but suffers from accuracy limitations in practical applications. The RCS is influenced by multiple factors, including vehicle shape, surface material, radar angle and wavelength, leading to inconsistent and often unreliable performance in vehicle classification. Elevation data hold significant potential for traffic detection and vehicle classification, yet current research has not fully exploited its application value. Therefore, leveraging elevation data for vehicle classification and detection not only overcomes the limitations of existing methods but also offers novel solutions for the traffic detection field.

## 3. Experimental Scenario

**Figure 1:**
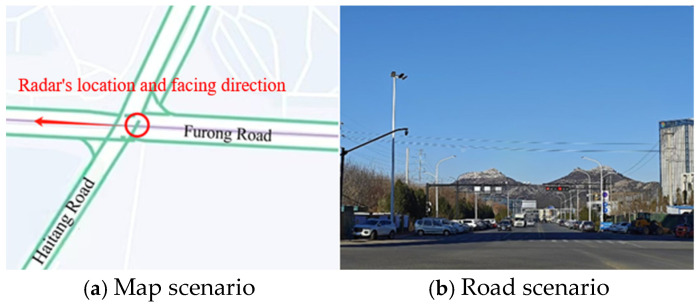
Experimental scenario.

The experimental scenario is located at the intersection of Furong Road and Haitang Road in Jinan City, Shandong Province, as shown in Figure 1. The radar is installed on the west side of the intersection for detecting the movement characteristics of vehicles from the north direction.

In this paper, the ARS548 mm wave radar is used for data collection and further analysis. The ARS548 [32] is part of the fifth-generation high-performance radar series of Continental, boasting 4D high-resolution imaging capabilities. This radar offers a higher angular resolution, longer detection range, and superior ability to measure the pitch angle of targets, significantly improving the detection and recognition accuracy for ITS. This radar offers a higher angular resolution, longer detection range, and superior ability to measure the pitch angle of targets, significantly improving detection and recognition accuracy for ITSs.

**Figure 2:**
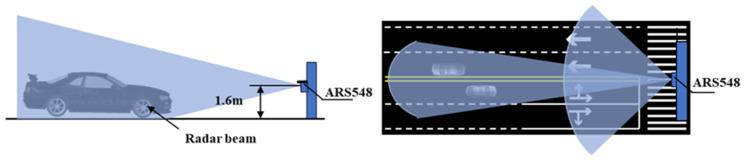
Radar installation scenario.

The vehicles are classified into small vehicles and large vehicles, in which the small vehicles include private car, taxi car, off-road vehicle, etc., and the large vehicles include transit, truck, dust car, etc. The radar is installed at 1.6 m above the ground, and further, the elevation angle and azimuth angle are configured for horizontal alignment to optimize its forward detection capabilities. This installation layout ensures that vehicles pass adjacent to the radar rather than directly underneath it. The installation layout of the radar sensor is shown in Figure 2.

In this paper, the wireshark is employed to capture and present the ARS548 mm-wave radar’s data. Referring to the SOME/IP protocol, the original radar data contain a header and a payload. The header includes the service ID and method ID, which are used to identify the service and data length. The payload mainly includes the target’s detailed data such as spatial position, speed and RCS, which are presented in Table 1. Additionally, the radar parameter configuration information is shown in Table 2.

## 4. Statistical Modeling of Elevation Data and Maximum Likelihood Classification Method

### 4.1. Statistical Modeling of Elevation Data

**Figure 3:**
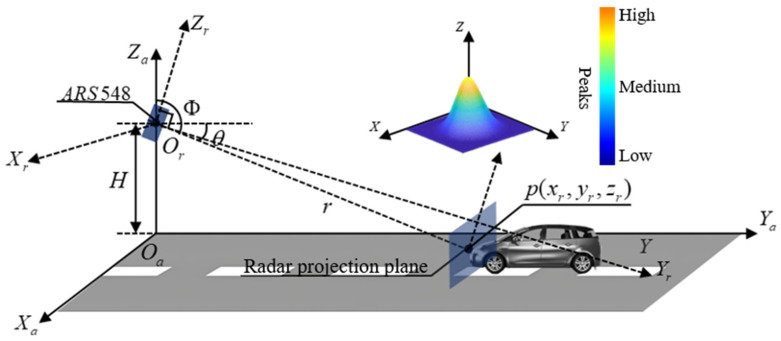
Radar reflection mathematical model and Gaussian plane.

Referring to the experimental scenario, the distribution of radar beams on the target plane (e.g., the horizontal plane) is as shown in Figure 3. In this paper, the actual road coordinate system $O_{a}-X_{a}Y_{a}Z_{a}$ and the radar coordinate system $O_{r}-X_{r}Y_{r}Z_{r}$ are established for further analysis, as shown in Figure 3.

In the $O_{r}-X_{r}Y_{r}Z_{r}$ coordinate system, the target’s position follows a three-dimensional Gaussian distribution referring to [33]. This is because the radar detects the target’s position using reflected beams, and the target’s reflection points typically have some spread, which can be modeled using a Gaussian distribution.

The radar is installed at a fixed height H with a pitch angle θ and an azimuth angle Φ. The transformation between the radar coordinate system and the actual road coordinate system can be accomplished through rotation and translation. The position of a point $P_{r}=(X_{r},Y_{r},Z_{r})$ in the $O_{r}-X_{r}Y_{r}Z_{r}$ can be transformed into the position $P_{a}=(X_{a},Y_{a},Z_{a})$ in the $O_{a}-X_{a}Y_{a}Z_{a}$ using a rotation matrix R and a translation vector $T=(T_{x},T_{y},T_{z})$ by Equation (1).

(1) $$P_{a}=R\;\cdot \;P_{r}+T,$$

where R is the rotation matrix, aligning the coordinate systems’ directions. T is the translation vector, which describes the relative position of the origins between the two coordinate systems.

Since the distribution of radar echo reflection points from a certain vehicle target follows a Gaussian distribution, the probability density function for the target’s reflection point $\left(X_{r},Y_{r},Z_{r}\right)$ in the $O_{r}-X_{r}Y_{r}Z_{r}$ can be calculated by Equation (2).

(2) $$f_{r}(X_{r},Y_{r},Z_{r})=\frac{1}{{\left(2\pi \right)}^{3/2}\sigma_{xr}\sigma_{yr}\sigma_{zr}}\;\cdot \;\exp(-\frac{{\left(X_{r}-\mu_{xr}\right)}^{2}}{2\sigma_{xr}^{2}}-\frac{{\left(Y_{r}-\mu_{yr}\right)}^{2}}{2\sigma_{yr}^{2}}-\frac{{\left(Z_{r}-\mu_{zr}\right)}^{2}}{2\sigma_{zr}^{2}}),$$

where $u_{xr},u_{yr},u_{zr}$ are the mean positions of the target. $\sigma_{xr},\sigma_{yr},\sigma_{zr}$ are the standard deviations in each direction, describing the spread of the target’s position.

The radar’s installation height defines the starting point of the radar beam, while the pitch angle θ determines the propagation angle of the beam in the vertical direction; hence, the radar’s height H and pitch angle θ directly affect the target’s position. If the target is at a distance r from the radar, the vertical position $Z_{r}$ of the target can be calculated by Equation (3).

(3) $$Z_{r}=H+r\;\cdot \;\sin(\theta ),$$

where r is the distance between the radar and the target.

The horizontal coordinates $X_{r}$ and $Y_{r}$ of the target are also affected by the pitch angle θ. Assuming Φ as the azimuth angle of the target, the horizontal position can be calculated by Equations (4) and (5).

(4) $$X_{r}=r\;\cdot \;\cos(\theta )\;\cdot \;\cos(\Phi ),$$

(5) $$Y_{r}=r\;\cdot \;\cos(\theta )\;\cdot \;\sin(\Phi ),$$

By applying the coordinate transformation, the target spatial position in the $O_{r}-X_{r}Y_{r}Z_{r}$ can be converted to the $O_{a}-X_{a}Y_{a}Z_{a}$. After transformation, the target’s position $\left(X_{a},Y_{a},Z_{a}\right)$ still follows a Gaussian distribution. Referring to Equation (1), the probability density function of the target in the $O_{a}-X_{a}Y_{a}Z_{a}$ can be described by Equation (6).

(6) $$f_{a}(X_{a},Y_{a},Z_{a})=f_{r}[R^{-1}\;\cdot \;(P_{a}-T)],$$

where $R^{-1}$ is the inverse of the rotation matrix. Based on the aforementioned analysis, the target’s position in $O_{a}-X_{a}Y_{a}Z_{a}$ still follows a Gaussian distribution.

The mean $u_{a}$ and the covariance matrix $\Sigma_{a}$ in the $O_{a}-X_{a}Y_{a}Z_{a}$ can be expressed by Equations (7) and (8), respectively.

(7) $$\mu_{a}=R\;\cdot \;\mu_{r}+T,$$

(8) $$\Sigma_{a}=R\;\cdot \;\Sigma_{r}+R^{T},$$

where $u_{r}$ is the mean in the $O_{r}-X_{r}Y_{r}Z_{r}$, and $\Sigma_{r}$ is the covariance matrix in $O_{r}-X_{r}Y_{r}Z_{r}$, containing the variances $\sigma_{xr},\sigma_{yr},\sigma_{zr}$.

The Gaussian distribution possesses the property of retaining its Gaussian form under linear transformations. Hence, the elevation value distribution of the target in the $O_{r}-X_{r}Y_{r}Z_{r}$ remains Gaussian characteristic after scaling and rotation transformations. The probability density function (PDF) of the target in the $O_{r}-X_{r}Y_{r}Z_{r}$ can be expressed by Equation (9).

(9) $$f_{r}(X_{r},Y_{r},Z_{r})=\frac{1}{{\left(2\pi \right)}^{3/2}{\left|\Sigma_{r}\right|}^{1/2}}\;\cdot \;\exp[-\frac{1}{2}{\left(P_{r}-\mu_{r}\right)}^{T}\Sigma_{r}^{-1}(P_{r}-\mu_{r})]$$

Under specific conditions of the radar installation at certain height, pitch angle and azimuth angle, the Gaussian distribution of the target can be determined. The geometric transformations affect only the mean and covariance matrix of the distribution, which in turn alters the shape and location of the distribution but not its fundamental Gaussian nature.

### 4.2. Maximum Likelihood Estimation Classification Model Based on Gaussian Distribution

Based on the distribution features of elevation data in different types of vehicles, this paper presents a vehicle type classification method using the maximum likelihood estimation (MLE). By conducting statistics on a large volume of data from different vehicle types, the respective mean vectors and covariance matrices can be acquired. For the trajectory data of each vehicle, the joint likelihood values of both the small and large vehicles can be calculated and expressed by Equation (10).

(10) $$L(w)=p(x_{1},x_{2},\cdots ,x_{N}|w)=p(x_{1}|w)p(x_{2}|w)\cdots p(x_{N}|w),$$

where w represents the model parameters, and $x_{1},x_{2},\cdots ,x_{N}$ are the data samples. The joint likelihood function L(w) represents the probability of observing the data at parameter w.

To simplify the calculation, we assume the errors $\xi_{i}=y_{i}-w^{T}x_{i}$ are normally distributed and expressed by Equation (11).

(11) $$\xi_{i}∼N(0,\sigma^{2})$$

The probability density function of $\xi_{i}$ is expressed by Equation (12).

(12) $$P(\xi_{i})=\frac{1}{\sqrt{2\pi \sigma^{2}}}e^{-\frac{\xi_{i}^{2}}{2\sigma^{2}}}$$

Substituting $\xi_{i}=y_{i}-w^{T}x_{i}$, the PDF for each data point is described by Equation (13).

(13) $$P(y_{i}|x_{i};w)=\frac{1}{\sqrt{2\pi \sigma^{2}}}e^{-\frac{{\left(y_{i}-w^{T}x_{i}\right)}^{2}}{2\sigma^{2}}}$$

Referring to Equations (10) and (13), the joint likelihood function for the entire dataset can be calculated by Equation (14).

(14) $$L(w)=\prod_{i=1}^{N}p(y_{i}|x_{i};w)=\prod_{i=1}^{N}\frac{1}{\sqrt{2\pi \sigma^{2}}}e^{-\frac{{\left(y_{i}-w^{T}x_{i}\right)}^{2}}{2\sigma^{2}}}$$

Taking the logarithm of Equation (14), the joint log-likelihood function can be expressed by Equation (15) and further simplified as Equation (16).

(15) $$\log L(w)=\sum_{i=1}^{N}\log\left(\frac{1}{\sqrt{2\pi \sigma^{2}}}e^{-\frac{{\left(y_{i}-w^{T}x_{i}\right)}^{2}}{2\sigma^{2}}}\right)$$

(16) $$\log L(w)=-\frac{N}{2}\log(2\pi \sigma^{2})-\frac{1}{2\sigma^{2}}\sum_{i=1}^{N}{\left(y_{i}-\omega^{T}x_{i}\right)}^{2}$$

In the training phase, the elevation data for small and large vehicles are fitted into two-dimensional Gaussian distributions, respectively. For each vehicle type, the mean vectors $\mu_{k}$ and covariance matrices $\Sigma_{k}$ can be calculated by Equations (17) and (18), respectively.

(17) $$\mu_{k}=\frac{1}{N_{k}}\sum_{i=1}^{N_{k}}X_{i},$$

(18) $$\Sigma_{k}=\frac{1}{N_{k}}\sum_{i=1}^{N_{k}}(X_{i}-\mu_{k}){\left(X_{i}-\mu_{k}\right)}^{T},$$

where $N_{k}$ is the number of data points.

During the training phase, the Gaussian distribution parameters for small and large vehicles can be determined by maximizing the joint likelihood function to obtain the optimal parameters for each vehicle type.

In the validation phase, if the elevation data of each vehicle are given, the joint log-likelihood values for different vehicle types can be calculated by Equation (19).

(19) $$\log L_{s,l}=\sum_{i=1}^{N}\log p(X_{i}|\mu_{s,l},\Sigma_{s,l})$$

Finally, the classification of vehicles can be determined according to the maximum joint log-likelihood values by Equation (19).

## 5. Experimental Results and Analysis

### 5.1. Elevation Data Feature Analysis

#### 5.1.1. Statistical Features of Overall Elevation Data

In complex and variable traffic environments, the raw data collected by millimeter-wave radar often contain missing or anomalous trajectory points due to system errors, sensor nonlinear responses and environmental noise. This paper screens, filters and corrects the original point cloud data to ensure data integrity and reliability.

**Figure 4:**
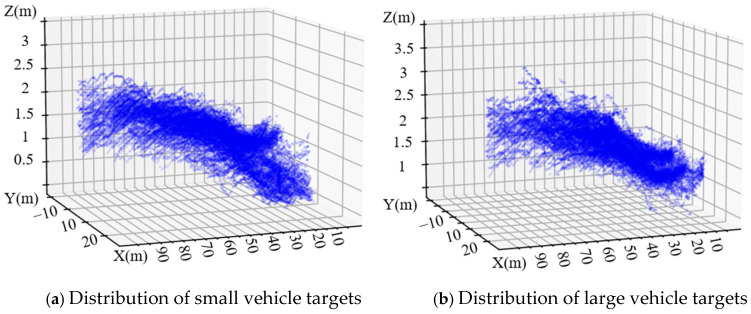
Point cloud distributions of vehicles by 4D radar.

Taking the center of the radar antenna as the coordinate original, the radar’s radial direction as the X-axis, the tangent direction as the Y-axis and the vertical direction as the Z-axis, the point cloud distributions of targets by the 4D radar are shown in Figure 4.

**Figure 5:**
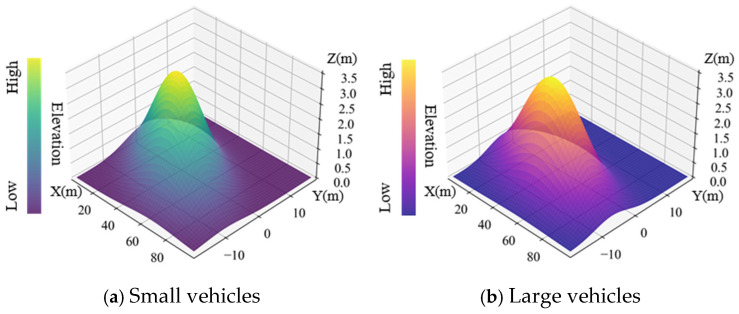
Gaussian distribution features of different vehicle types.

Referring to Figure 4, it is evident that the elevation presents a growth trend while the radial distance increases. The reason is that in the experimental scenario, the road surface is upwards and the height increases. To more intuitively display the spatial distribution characteristics of small and large vehicles, the three-dimensional probability density distribution maps are shown in Figure 5.

For Figure 5, the Gaussian fitting results for small vehicles and large vehicles are expressed by Equation (20).

(20) $$f_{s,l}(x,y,z)=\frac{1}{{\left(2\pi \right)}^{3/2}\sqrt{\left|\Sigma \right|}}\exp(-\frac{1}{2}{\left[\begin{array}{c} x-\mu_{x}\\ \begin{array}{l} y-\mu_{y}\\ z-\mu_{z} \end{array} \end{array}\right]}^{T}\Sigma^{-1}\left[\begin{array}{c} x-\mu_{x}\\ \begin{array}{l} y-\mu_{y}\\ z-\mu_{z} \end{array} \end{array}\right])$$

In Equation (20):

(21) $$\mu_{s}=\left[\begin{array}{c} 45.3\\ -2.97\\ 1.36 \end{array}\right],\mu_{l}=\left[\begin{array}{c} 49.34\\ -4.61\\ 1.82 \end{array}\right],$$

(22) $$\Sigma_{s}=\left[\begin{array}{ccc} 478.47 & -57.25 & 6.95\\ -57.25 & 41.41 & -1.92\\ 6.95 & -1.92 & 0.20 \end{array}\right],\;\Sigma_{l}=\left[\begin{array}{ccc} 545.50 & -18.60 & 9.30\\ -18.60 & 25.40 & -0.01\\ 9.30 & -0.01 & 0.24 \end{array}\right].$$

From Figure 5, it is evident that the elevation exhibits significant Gaussian distribution characteristics, and it shows obvious differences between small and large vehicles. The distribution of small vehicles is more concentrated, while large vehicles have a wider distribution range, especially in radial distance and elevation directions.

**Figure 6:**
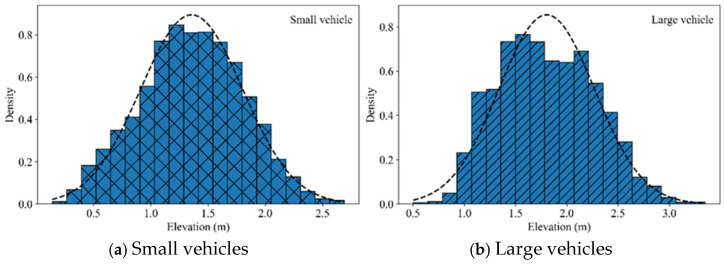
Elevation histograms and Gaussian fitting curves for small (**a**) and large (**b**) vehicles.

Statistical analysis on all the collected elevation data has been made and the elevation histograms and probability density curve function for both the small and large vehicles has been generated with the results shown in Figure 6.

In Figure 6, the PDF for small vehicles and large vehicles are calculated and expressed by Equations (23) and (24), respectively.

(23) $$f_{s}(x)=\frac{1}{\sqrt{2\pi \;\cdot \;0.02}}\cdot \exp(-\frac{{\left(x-1.36\right)}^{2}}{2\;\cdot \;0.02})$$

(24) $$f_{l}(x)=\frac{1}{\sqrt{2\pi \;\cdot \;0.22}}\;\cdot \;\exp(-\frac{{\left(x-1.80\right)}^{2}}{2\;\cdot \;0.22})$$

The fitting goodness for the small vehicle is 0.98, while that for the large vehicle is 0.91. Since the fitting goodness values are close to 1, the fitting results for both the small and large vehicles are satisfactory.

In Figure 6, the elevation data distribution of small vehicles is concentrated and stable, while that of large vehicles is more dispersed, especially over a longer distance where the data variability increases significantly. These results are consistent with the conclusions acquired by the aforementioned content.

#### 5.1.2. Statistical Features of Cross-Section Elevation Data

**Figure 7:**
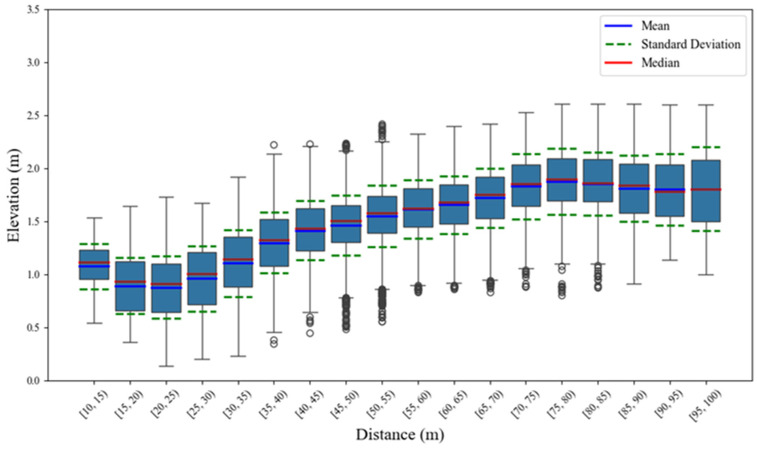
Distribution characteristics of elevation features for small vehicles.

**Figure 8:**
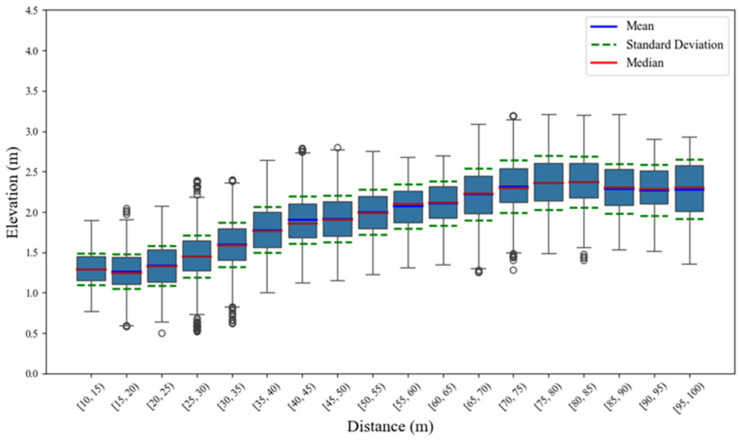
Distribution characteristics of elevation features for large vehicles.

In order to further excavate the features of the 4D elevation data, this paper conducts statistical analysis on the elevation data under different cross-sections. The statistical distributions of the overall elevation data of two types of vehicles are shown in Figure 7 and Figure 8, respectively.

And the statistical parameters are presented in Table 3 and Table 4, respectively.

Referring to Figure 7 and Figure 8, and Table 3 and Table 4, the elevation distribution center (mean and median) and dispersion (standard deviation) of large vehicles are higher than those of small vehicles, indicating that large vehicles are more widely distributed while small vehicles are more concentrated. Moreover, it is evident that the mean value, standard deviation, and median and interquartile range under different horizontal distances present growth trends while the distance increases. The reason is that with the distance increasing, the RCS of the target becomes smaller, further inducing the effective echo reflection points to become sparse and present more discrete features.

**Figure 9:**
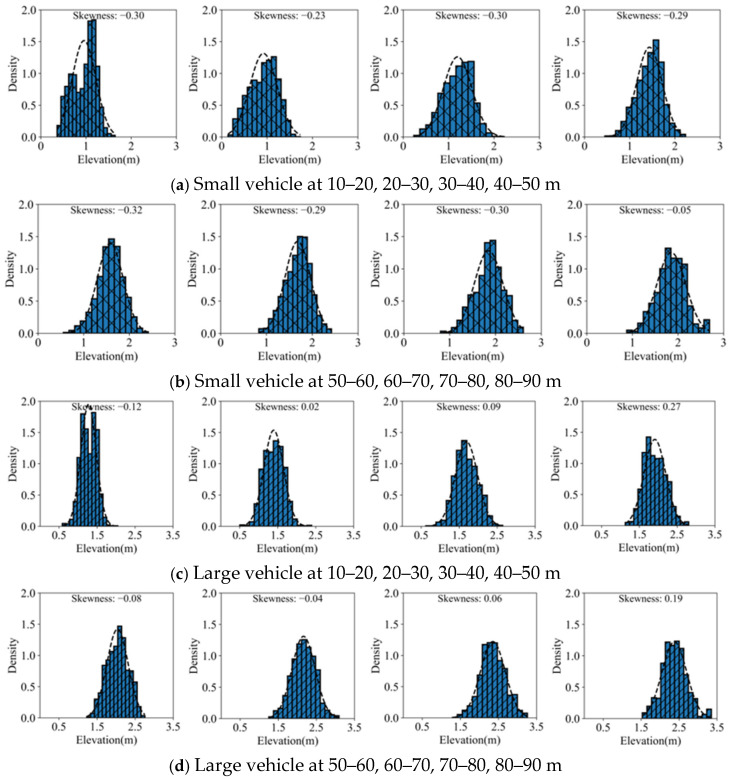
Elevation probability distributions for two vehicle types at different cross-sections.

Taking horizontal distances of 10–20 m, 20–30 m, 30–40 m, 40–50 m, 50–60 m, 60–70 m, 70–80 m, and 80–90 m as examples, the probability distributions of cross-sectional elevation values are shown in Figure 9.

**Figure 10:**
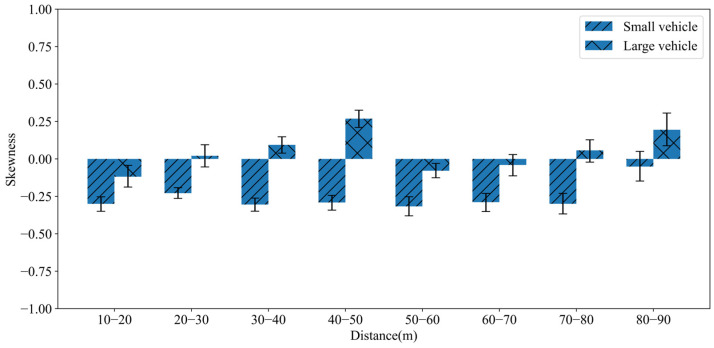
Skewness coefficients for small and large vehicles.

From Figure 9, it can be observed that the probability distribution of cross-section elevation values generally follows a normal distribution. However, it still exhibits skewness to a certain extent. For small vehicles, the skewness coefficients are generally less than 0, indicating a right-skewed distribution. While for large vehicles, the skewness coefficients are mostly greater than 0, indicating a left-skewed distribution. This skewness coefficients are further presented in Figure 10.

This result is primarily influenced by the installation height and pitch angle of the radar equipment as well as the height differences between the two types of vehicles. Under the installation conditions of the experimental scenario in this paper, the effective echo reflection points are mainly concentrated below the center position for small vehicles. While for large vehicles, the effective echo reflection points are primarily concentrated above the center position. However, due to the broader range of large vehicles, situations may arise where small vehicles are inadvertently included.

### 5.2. Vehicle Type Classification Results Based on Maximum Likelihood Estimation

The above analysis of elevation features for small and large vehicles is intended to provide sufficient data characteristics for vehicle classification. The original data are divided into a training set and a validation set. Each sample in the training set is labeled into large or small vehicle for further optimizing the parameters for maximum likelihood estimation, including the mean vector and covariance matrix. The samples in the validation set are unlabeled and used to evaluate the model’s performance, including accuracy, precision and recall.

By comparing the log-likelihood values of the two models, the classification of each sample is acquired. Using the aforementioned sample set, the classification results of the proposed method achieve the accuracy of 0.92, precision of 0.87 and recall of 0.98. The results show that this method effectively captures the variability differences in the elevation data distributions of large and small vehicles. However, the precision is slightly lower than the recall. The reason is that parts of small vehicles are misclassified as large vehicles. This is because small vehicle data can easily fall into the distribution of the large vehicle, which has a much broader distribution range.

**Figure 11:**
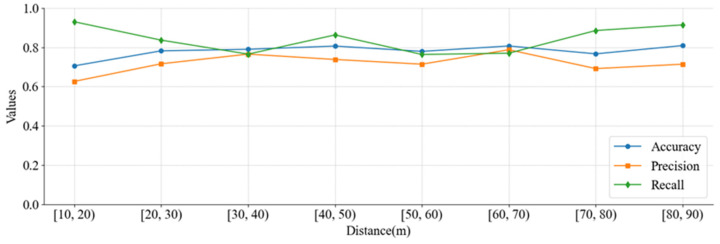
MLE-based classification results of vehicle types at different cross-sections.

To further investigate the classification performance of different vehicles, the accuracy, precision and recall values at different cross-sections per 10 m are shown in Figure 11. The results highlight consistent good performance at most sections with some minor variations in precision. These variations can be attributed to overlapping characteristics in small and large vehicle distributions.

**Figure 12:**
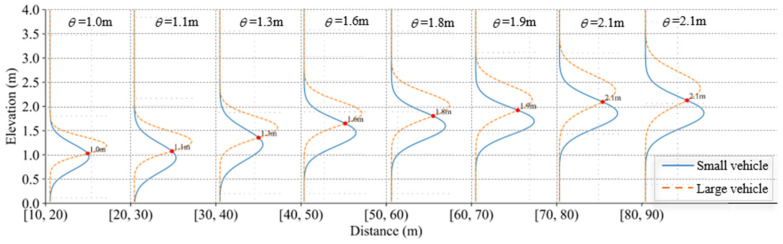
Elevation threshold values for different cross-sections.

To further validate the experimental results, the proposed method is compared with the traditional threshold-based method. The elevation threshold for the classification of large and small vehicles is defined as the intersection point of the two types of data distribution curves, which are denoted as θ. For different cross-sections, the data distribution curves and the elevation threshold values are presented in Figure 12.

**Figure 13:**
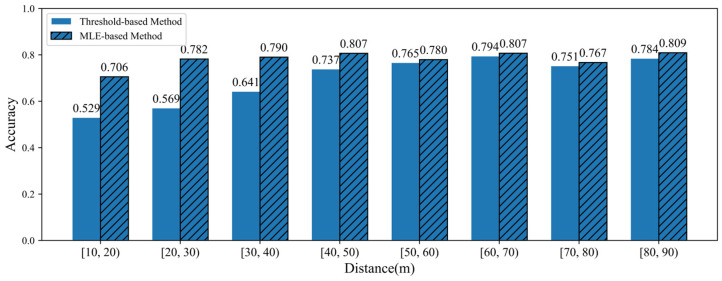
Accuracy comparison between MLE-based method and threshold-based method.

Referring to Figure 12, the results of the accuracy performance between the MLE-based method and threshold-based method are shown in Figure 13.

Referring to Figure 12 and Figure 13, it is obvious that when marked variations in elevation distributions of different vehicle types are detected at a far distance, the threshold-based method demonstrates effective results. However, the MLE-based method achieves better classification performance for different cross-sections compared with the threshold-based method. With closing distance, the proposed method demonstrates a growing superiority in classification accuracy with the performance gap expanding significantly. The reason is that the MLE-based method constructs a likelihood function to quantify how well the parameters fit the observed elevation data. It uses the conditional probability distribution of hypothesized data to build a probabilistic relationship between input elevation features and the vehicle classifications. Compared to the threshold-based method, the MLE-based method can directly reflect the influence of evaluation features on the classification probability, consequently achieving higher accuracy.

The cross-sectional results, combined with the overall performance metrics, emphasize the effective of the classification method in distinguishing large and small vehicles. This approach not only validates the model’s effectiveness but also provides insights into potential optimization strategies, paving the way for enhanced classification accuracy and reliability.

## 6. Conclusions and Future Work

This paper explores the elevation features of vehicles collected by 4D millimeter-wave radar and presents significant differences between small and large vehicles. It is obvious that large vehicles exhibit higher elevation values and a wider range of distribution with a left-skewed distribution in different cross-sections, while small vehicles are more concentrated with a right-skewed distribution. Moreover, the Gaussian-based maximum likelihood estimation model is applied for vehicle classification with an accuracy of 0.92, precision of 0.87 and recall of 0.98. The results show that the elevation from 4D millimeter-wave radar can serve as a significant feature for further vehicle classification, offering significant support for traffic monitoring the corresponding applications.

The experiment in this paper is carried out on a specific intersection where the vehicle dataset is insufficient. For future work, more cases will be experimented with for extending the dataset. Additionally, more supervised learning methods will be used for further study to obtain more accurate vehicle-type classification results.

## Figures and Tables

**Table 1:** Details of 4D millimeter-wave radar data output.

Category	Unit
Distance with reference to vehicle rear axle (X, Y, Z)	m
Relative and absolute velocity (X, Y)	m/s
Relative and absolute acceleration (X, Y)	m^2^/s
Width, length, leading	m
Radar cross-section	dB
RCS existence probability	-
Dynamic property (moving, stationary)	-

**Table 2:** Parameter configuration information of 4D millimeter-wave radar.

Category	Range
Resolution distance measuring	0~0.22 m
Azimuth/Elevation resolution	−0.1~0.1°
Detection azimuth angle	−90~90°
Detection elevation angle	−90~90°
Detection radial distance	−100~1600 m
Detection radial velocity	−120~120 m/s
Detection RCS	−128~127 dBm^2^
Object X coordinate	−100~1600 m
Object Y coordinate	−1600~1600 m
Object Z coordinate	−1600~1600 m

**Table 3:** Statistical parameters of small vehicles.

Horizontal Distance	Mean Value	Standard Deviation	Median Value	Interquartile Range
[10, 20)	0.97	0.07	0.20	1.0
[20, 30)	0.92	0.09	0.30	0.95
[30, 40)	1.20	0.10	0.32	1.23
[40, 50)	1.44	0.08	0.28	1.46
[50, 60)	1.58	0.08	0.29	1.59
[60, 70)	1.68	0.08	0.28	1.71
[70, 80)	1.85	0.10	0.31	1.87
[80, 90)	1.85	0.10	0.32	1.86

**Table 4:** Statistical parameters of large vehicles.

Horizontal Distance	Mean Value	Standard Deviation	Median Value	Interquartile Range
[10, 20)	1.28	0.04	0.20	1.27
[20, 30)	1.39	0.06	0.26	1.40
[30, 40)	1.69	0.08	0.30	1.67
[40, 50)	1.91	0.08	0.29	1.88
[50, 60)	2.04	0.08	0.28	2.06
[60, 70)	2.16	0.09	0.30	2.17
[70, 80)	2.35	0.11	0.33	2.34
[80, 90)	2.38	0.11	0.33	2.37

## Data Availability

The original contributions presented in this study are included in the article. Further inquiries can be directed to the corresponding author(s).
